# Oxyntic Gland Adenoma in a Patient With Refractory Reflux

**DOI:** 10.7759/cureus.38577

**Published:** 2023-05-05

**Authors:** Jolie Krooks, Harshwardhan Thaker, Suimin Qiu, Gabriel Reep, Jing He

**Affiliations:** 1 Pathology, University of Texas Medical Branch, Galveston, USA; 2 Gastroenterology and Hepatology, University of Texas Medical Branch, Galveston, USA

**Keywords:** oxyntic mucosa, fundic-gland type, gastric adenocarcinoma, chief cells, oxyntic gland adenoma

## Abstract

A 58-year-old African American male was referred for endoscopic evaluation due to a persistent nine-year history of reflux. Previous endoscopy nine years ago revealed a small hiatal hernia and chronic gastritis caused by *Helicobacter pylori (H. pylori)*, which was treated with triple therapy. During the current endoscopic evaluation, findings consistent with reflux esophagitis were identified, along with the discovery of an incidental 6 mm sessile polyp in the gastric fundus. Pathological examination revealed the presence of an oxyntic gland adenoma (OGA). Otherwise, the stomach was found to be unremarkable endoscopically and histologically.

OGA is a rare gastric neoplasm that is primarily observed in Japan, with very few reported cases in North America. Studies have suggested a potential association with antacids, while the role of *H. pylori* in the development of OGA remains controversial. Our patient’s OGA was completely resected during the endoscopy, with no recurrence noted on the three-month follow-up.

## Introduction

Oxyntic gland adenoma (OGA) is a relatively new entity that has undergone changes in nomenclature. As the name suggests, it is a relatively benign epithelial neoplasm that is comprised of chief cells and/or parietal cells with mild nuclear atypia [1,2]. Most recently, according to the 2019 World Health Organization (WHO) Classification of Gastric Carcinomas (5th edition), OGA is distinguished from gastric adenocarcinoma of fundic gland type (GA-FG) by the absence of submucosal invasion [3], which occurs in approximately half of all cases [4]. 

OGA is a rare neoplasm with an estimated prevalence of up to 0.36% in Japan. This estimation is based on the findings of a large retrospective study of 13,240 upper gastrointestinal endoscopies performed on 7,488 patients at an outpatient internal medicine and endoscopy clinic in Japan (Asahara Clinic, Akashi, Japan). Of note, the authors of the study note that their estimated period prevalence is much larger than previously reported [4]. Furthermore, cases of OGA reported outside of Japan are exceptionally scarce [5]. To the best of our knowledge, there have been less than 25 cases reported in the United States [6-9].

In this report, we present a case of OGA in a 58-year-old African American male with a history of previously treated *Helicobacter pylori (H. pylori) *infection. He was referred to our institution for endoscopic evaluation due to a nine-year history of reflux-associated symptoms refractory to antacid therapy. Although proton pump inhibitors (PPIs) had been prescribed, potential incompliance was noted in the electronic medical record. The patient also reported chronic use of baclofen with some improvement in symptoms.

Although cases of OGA have been described in association with PPIs and antihistamines, this association may be related to their high utilization in patients with reflux and *H. pylori *gastritis. OGA is typically discovered incidentally. Patients with reflux and* H. Pylori* gastritis often seek endoscopic evaluation; thus, OGA is more likely to be discovered in these patients. The roles of *H. pylori *in OGA pathogenesis and in mediating patients’ presentations are still controversial. Our patient had no endoscopic or pathologic evidence of persistent infection.

## Case presentation

A 58-year-old African American male patient was referred for endoscopy due to a nine-year history of intractable reflux-associated symptoms, which had recently worsened in severity. Notably, he had undergone upper endoscopy nine years ago, which revealed evidence of a small hiatal hernia and chronic gastritis secondary to *H. pylori*, for which he was prescribed triple therapy. Since the onset of symptoms, the patient had inconsistently taken prochlorperazine, omeprazole, and pantoprazole without improvement in symptoms. Baclofen was the only medication the patient took consistently.

**Figure 1:**
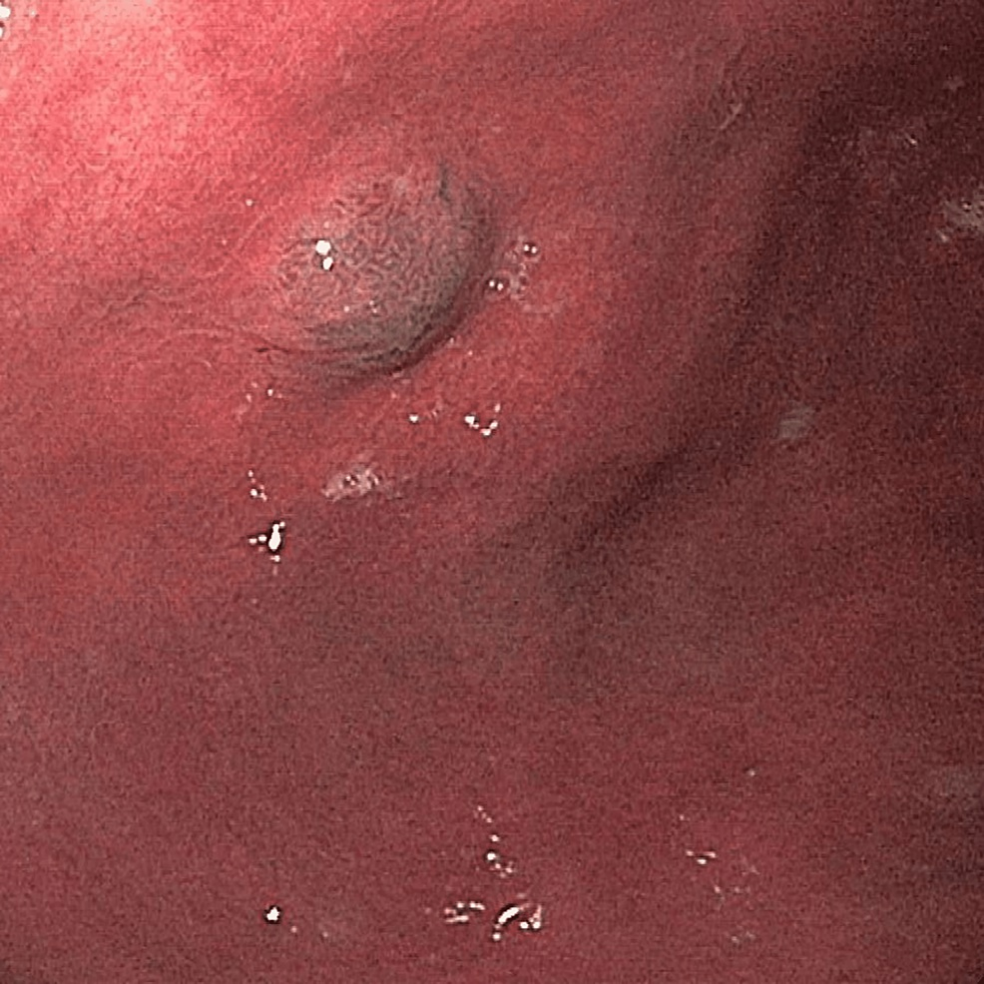
Endoscopic imaging reveals a small sessile lesion at the upper third of the gastric fundus with dilated vessels visualized with narrow-band imaging.

During endoscopy, a 6 mm sessile polyp was identified in the gastric fundus without any signs of recent bleeding (Figure 1). The rest of the stomach appeared normal. In the distal esophagus, LA Grade A reflux esophagitis and a medium-sized hiatal hernia were observed. The gastric polyp was completely resected by cold snare polypectomy.

**Figure 2:**
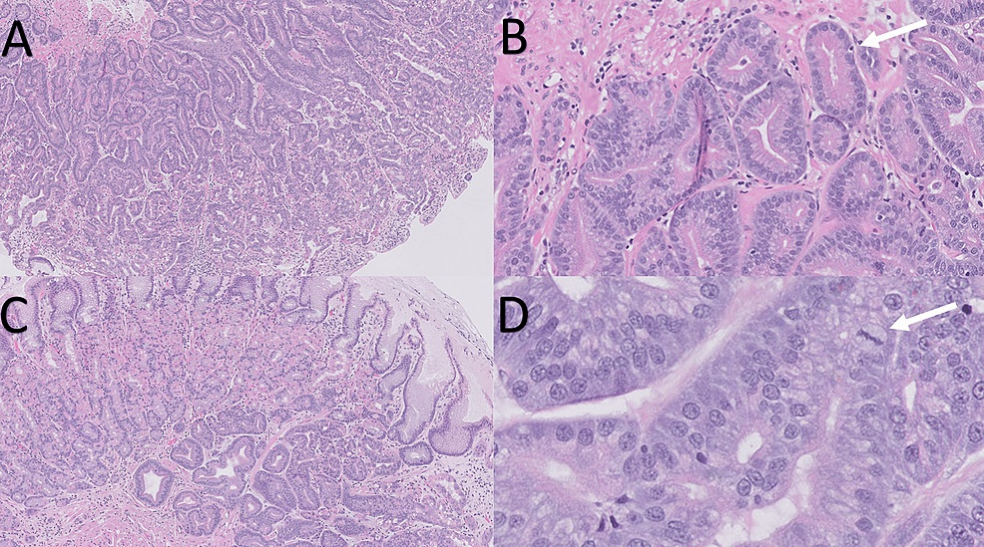
Histological findings of oxyntic gland adenoma (A) Hematoxylin and eosin staining shows a proliferation of branching glands composed predominantly of columnar cells with basophilic cytoplasm located in the deep portion of oxyntic mucosa (20x magnification). (B) Focally, there are glands comprised of parietal cells with eosinophilic granular cytoplasm (100x magnification). (C) There is a sharp demarcation between the lesion and normal gastric mucosa. The overlying surface foveolar epithelium is normal (50x magnification). (D) The lesion only has one mitotic figure (200x magnification).

Histological examination of the gastric polyp showed a neoplastic glandular proliferation with branching and anastomosing architecture. The neoplastic cells displayed characteristics of chief cell differentiation, with basally oriented round nuclei and basophilic cytoplasm. The nuclei appeared slightly enlarged and hyperchromatic with inconspicuous nucleoli (Figure 2A). Additionally, a minor component of parietal cells with eosinophilic granular cytoplasm was observed in some areas (Figure 2B). The lesion was well demarcated from the surrounding normal gastric mucosa. The overlying surface epithelium was also normal (Figure 2C). Mitotic activity was low, with only one mitotic figure identified in the entire specimen (Figure 2D). Pathologic evaluation of the submucosa was limited due to the superficial nature of the specimen. We did not identify background gastritis, atrophy, intestinal metaplasia, or *H. pylori*-like organisms, nor did we detect any areas of necrosis or lymphovascular invasion.

On three-month follow-up, endoscopic evaluation showed a scar in the gastric fundus where the polyp had been resected. There was no evidence of a residual lesion either endoscopically or histologically. 

## Discussion

In 2021, Ueyama et al. proposed a new classification called gastric epithelial neoplasm of fundic gland mucosa lineage (GEN-FGML), which includes OGA, GA-FG, and gastric adenocarcinoma of fundic-gland mucosa type (GA-FGM) [2]. Both OGA and GA-FG are types of low-grade epithelial neoplasms consisting of fundic gland-like cells that form architecturally irregular glands, typically covered by normal surface foveolar epithelium [2]. Conversely, GA-FGM is comprised of both fundic gland-like and foveolar-like cells, displays aggressive biologic behavior, and is considered a high-grade epithelial neoplasm [2]. Specifically, there is marked cytologic atypia or architectural distortion, substantial submucosal invasion, and lymphovascular invasion, which are typically only observed with GA-FGM [1-2]. While less common, GA-FGM still accounts for a significant proportion (25%) of GEN-FGML [2]. Although not yet reported, if an intramucosal tumor has severe cytological and/or architectural abnormality and invades the lamina propria, it would be classified as intramucosal adenocarcinoma of fundic gland type [1]. 

OGA is usually diagnosed in older adults (mean age 66 years) and has a slight male predilection [1,10]. Endoscopically, OGA manifests as a small raised subepithelial lesion (77%) with prominent dilated vessels on the surface, particularly in low-grade tumors with typical histologic features [1,2]. The mean tumor size is 5.9 mm, which is significantly smaller than the size of tumors with significant atypia and submucosal invasion (mean 26.8 mm) [1]. Central depression and increased size are increasingly recognized in GA-FG and GA-FGM, respectively [1,2].

A recent study on 165 patients investigated whether there are distinct endoscopic findings in patients with a current or prior history of *H. pylori* infection in comparison to those without the infection. The study revealed that *H. pylori *infection was significantly associated with tumors that were flat or in atypical locations of the stomach [11]. However, in our study, the patient presented with a typical lesion despite his prior *H. pylori *infection status. 

Histologically, OGA is comprised of cells that resemble those of normal oxyntic mucosa, generally with only mild cytologic atypia characterized by slight nuclear enlargement (5-9 μm). Architecturally, the cells form irregular branching, anastomosing glands. The tumor is located within the deep mucosa and covered by normal surface foveolar epithelium, which is consistent with the location of the chief cells. The tumor grows with a pushing border into the muscularis mucosae. Significant nuclear atypia, mitotic activity, and desmoplasia are absent [1,2,7].

Various patterns have been described. The chief cell-predominant pattern is by far the most common (96%-99%) [10,12] and consists of highly differentiated columnar cells with either pale blue or eosinophilic cytoplasm in the typical or eosinophilic variants, respectively. Other patterns include tumors with both chief cells and parietal cells, known as the mixed cell pattern, parietal cell-predominant pattern, as well as tumors containing both the chief cell-predominant and mixed cell patterns [1,10].

Though not necessary in our case, additional ancillary immunohistochemical staining can further support the diagnosis. Pepsinogen I, pepsinogen II, and runt related transcription factor 3 (RUNX3) can stain chief cells, but pepsinogen II is relatively nonspecific and also stains pyloric gland cells and mucus neck cells. H+/K+-ATPase, platelet-derived growth factor α (PDFRA-α), and human milk fat globule-2 (HMFG-2) can stain parietal cells and the staining may be focal. MUC6 staining, which is positive in pyloric gland cells and mucus neck cells, is positive in OGA, GA-FG, and GA-FGM. MUC5AC stains superficial foveolar epithelial cells and is only positive in GA-FGM. Markers of intestinal differentiation (CD10, CDX2, MUC2) are negative. While synaptophysin and CD56 may be positive, the tumor should not be mistaken for a neuroendocrine tumor as the diagnosis is based on morphology and these stains are non-specific [2,8,10].

OGA should be distinguished from other proliferative gastric epithelial lesions. Approximately 90% of gastric polyps are benign fundic gland polyps (FGPs) and hyperplastic polyps, whereas the remaining 10% are adenomas [13]. According to the WHO 2019 classification, there are four types of adenomas, including intestinal-type adenomas, foveolar-type adenomas, pyloric gland adenomas (PGAs), and OGAs [14]. Intestinal and foveolar adenomas arise from the surface epithelium (either metaplastic or native, respectively) and are the most prevalent of the adenomas, accounting for 56% and 41% of cases, respectively. Conversely, PGAs and OGAs arise from the deep glandular compartment, with OGAs are predominantly composed of parietal and chief cells and PGAs are composed of mucous neck cells [13]. 

Infection with *H. pylori *is the primary risk factor in the development of gastric cancer, with approximately 90% of patients having a current or prior history of infection [15]. *H. pylori *infection-associated gastric carcinomas typically involve the distal third of the stomach. The progression from *H. pylori* infection to carcinoma occurs through Correa’s cascade, in which inflammation leads to metaplasia, dysplasia, and finally carcinoma [14,16]. Therefore, atrophic and/or metaplastic changes are frequently identified in the background gastric mucosa.

*H. pylori *does not seem to contribute to the development of gastric-type adenocarcinomas, which include foveolar, pyloric, and fundic-gland type related adenocarcinomas [4,17]. In contrast to H. pylori infection-associated gastric carcinomas, most OGA/GA-FG involve the upper third of the stomach (80%). They are less frequently observed in the middle third (18%) and lower third (1%) of the stomach [10]. Furthermore, regardless of infection status, 90% of OGA are identified in non-atrophic gastric mucosa without surrounding inflammation or metaplasia in background mucosa [4]. 

Some cases of OGA have been noted adjacent to gastric mucosa with PPI-associated histologic changes, such as FGPs and parietal cell hyperplasia [10]. Notably, PPI use has been identified in up to 37% of patients diagnosed with OGA, with the median duration of use being 646 days (range: 35-5,381 days) [4]. Among these patients with PPI use, FGPs are identified concurrently in 33% and multiple OGA are identified in 11% [4]. The role of PPIs in the development of FGPs has been extensively studied due to their increased prevalence. Similar to OGA, FGPs usually develop in non-atrophic oxyntic mucosa and are comprised of cystically dilated oxyntic glands lined by parietal cells, chief cells, and potentially mucous neck cells. The overlying surface epithelium is typically normal. Prolonged PPI use is associated with a four to five times increased risk of FGP [18]. The increased prevalence of FGPs parallels the rising usage of PPIs [18]. Acid suppression resulting from PPI use leads to excess serum gastrin, a downstream target of Wnt signalling that stimulates the growth of oxyntic mucosa. FGPs are also inversely associated with *H. pylori* infection. It is speculated that *H. pylori* prevents cystic dilation of glands resulting from impaired gastric secretions through the degradation of gastric mucus [18].

While these findings suggest that acid suppression therapy may contribute to the pathogenesis of OGA, an alternative explanation is that OGAs are asymptomatic and typically found incidentally during endoscopic evaluation for reflux. Concurrent use of antacid therapy may be coincidental and unrelated to OGA pathogenesis.

Both gastrointestinal endoscopic mucosal resection and submucosal dissection are highly effective in completely removing the OGAs, with success rates of 92% and 100%, respectively, and low recurrence rates [4].

## Conclusions

In summary, we present a rare case of OGA discovered incidentally in an African American male undergoing evaluation for reflux-associated symptoms. The patient had previously been treated for *H. pylori* with triple therapy. As the prevalence of reflux continues to increase in the Western world, we may see a rise in the number of reported OGAs due to their incidental discovery with screening endoscopies. More studies are needed to ascertain whether PPIs contribute to the pathogenesis of OGA or whether the association between PPIs and OGAs is noncausal and merely due to the frequent utilization of PPIs in treating reflux. Furthermore, more studies are needed to investigate whether OGAs in North America behave similarly to those in Japan. Our patient’s lesion was completely resected with no evidence of recurrence on three-month follow-up.
